# A Comprehensive Review: Genetic Mapping of Genes Associated with Green Leaf Color Variations in Main Vegetable Crops

**DOI:** 10.3390/plants14111609

**Published:** 2025-05-25

**Authors:** Menghao Wang, Xinyin Wang, Yue Wang, Xiyue Yang, Xiabing Li, Junrong Chen, Shengjun Feng

**Affiliations:** 1 Collaborative Innovation Center for Efficient and Green Production of Agriculture in Mountainous Areas of Zhejiang Province, College of Horticulture Science, Zhejiang Agriculture and Forestry University, Hangzhou 311300, China; 2022114011023@stu.zafu.edu.cn (M.W.); h2022614022078@stu.zafu.edu.cn (X.W.); wy9127@stu.zafu.edu.cn (Y.W.); 202101070335@stu.zafu.edu.cn (X.Y.); lxb6225@stu.zafu.edu.cn (X.L.); junrongchen@stu.zafu.edu.cn (J.C.); 2 College of Advanced Agricultural Sciences, Zhejiang Wanli University, Ningbo 315100, China

**Keywords:** leaf color mutation, gene mapping, molecular breeding

## Abstract

The diversity in the coloration of vegetable leaves carries substantial commercial and nutritional importance. Investigating mutants of vegetable leaf color is of paramount importance for uncovering the mechanisms behind leaf color variation and for developing new high-yielding vegetable varieties with enhanced photosynthetic efficiency. This paper encapsulates the principal advancements in research concerning vegetable leaf color mutants, elucidates the origins and gene mapping techniques for these mutants, and delineates the molecular regulatory mechanisms involved in leaf color transformation. The findings presented serve as a valuable reference for the cultivation of superior plant material and for fostering the sustainable growth of vegetable cultivation practices.

## 1. Introduction

Leaf color is a critical agronomic trait in vegetable crops, with green leaves being essential for efficient photosynthesis and overall plant health. While most vegetable crops exhibit green leaves, genetic mutations that disrupt chloroplast development or inhibit chlorophyll synthesis can lead to leaf color variations, resulting in a spectrum of leaf colors. These leaf color mutants, characterized by distinct and easily identifiable traits, occur relatively frequently in higher plants and often manifest during the seedling stage. Awan et al. have categorized leaf color mutations into eight types: albino, greenish-white, white emerald, light green, greenish-yellow, etiolation, yellow-green, and striped [1]. These mutants can also be classified based on differences in chlorophyll content, such as total increase or deficiency types, and further divided into chlorophyll a or b deficiency types. Environmental factors, including temperature and light conditions, also influence leaf color, leading to varying phenotypes. Leaf color mutants often affect the photosynthetic rate of plants, impacting their growth, development, and agricultural productivity. They are ideal models for studying the photosynthetic mechanisms of vegetables and have shown increasing research value and potential applications. Recent advances in genetic mapping techniques, such as map-based cloning, Bulked Segregant Analysis (BSA), and transcriptome sequencing, have enabled the identification of genes associated with leaf color variations in various vegetable crops. These techniques have been applied to a range of vegetables, including tomatoes [2,3,4], cucumbers [5,6,7], watermelons [8], eggplants [9], cabbages [10,11], Chinese cabbages [12], phaseolus vulgaris [13], and peppers [14,15]. This comprehensive review aims to provide an up-to-date overview of the genetic mapping of genes associated with green leaf color variations in main vegetable crops. It highlights the key genes and loci identified through these advanced techniques and discusses their roles in chloroplast development, chlorophyll biosynthesis, and degradation. By summarizing the current research progress and identifying gaps for future studies, this review seeks to contribute to the development of vegetable varieties with improved leaf color traits, thereby enhancing crop productivity and quality.

## 2. Source of Leaf Color Mutation in Vegetables

### 2.1. Spontaneous Mutation

Spontaneous mutations in vegetable crops are often triggered by significant changes in natural conditions, such as light, temperature, and soil. These mutations primarily involve alterations in single genes. Although the probability of such mutations occurring is relatively low, they can be directly utilized in traditional breeding programs. To date, numerous natural mutants have been documented across various vegetable crops. For instance, Yan et al. identified a spontaneous mutant from the cucumber inbred line g32, which exhibits light-sensitive albinism [16]. Another example is the GL mutant with deep green leaves, isolated from a spontaneous mutation in the tomato inbred line “716” [17]. Additionally, a spontaneous mutant yl20, characterized by yellow cotyledons and young leaves displaying two phenotypes—yellow and light green—was derived from the wild-type green leaf eggplant [9].

### 2.2. Artificial Mutagenesis

Artificial mutagenesis, known for its high mutation efficiency, is currently the primary method for generating vegetable leaf color mutants. This encompasses a range of techniques, including chemical mutagenesis, physical mutagenesis, T-DNA insertion, and others. The relatively simple types of gene mutations induced by artificial mutagenesis make the resulting mutations easier to identify; thus, this approach is widely used in gene localization. Ethyl methane sulfonate (EMS) mutagenesis is a key method in chemical mutagenesis and has been frequently employed to obtain vegetable leaf color mutant materials. For instance, Song and colleagues derived the cucumber yellow young leaf mutant “vyl” through EMS mutagenesis. This mutant exhibited virescent yellow leaves at the seedling stage, which gradually turned green as the leaves matured. The hybridization of this mutant revealed a segregation ratio of 3:1 in its F*2* generation, suggesting that the trait is controlled by a single recessive gene [18]. In cabbage, Zhao and his team also identified a mutant similar to “vyl”, with young leaves showing a light yellow phenotype that transitioned to pale green upon maturity, named “yvl” [19]. Zhang and co-authors obtained a cucumber virescent mutant through EMS mutagenesis, where the cotyledons and the first through fifth true leaves of this mutant were initially yellow, gradually turning green, while the sixth true leaf was normal green. Compared to the wild type, the adult plants of the mutant were dwarfed, with slower growth rates and delayed flowering times [20]. Xiong and associates obtained the cucumber yellow leaf mutant “yl2.1” via EMS mutagenesis, with the mutant’s cotyledons being yellow after germination. The leaf color of “yl2.1” during the first true leaf stage of seedling growth was light green. As the plant progressed to the second and third true leaf stages, the leaf color of “yl2.1” gradually darkened to yellow, without turning green as the leaves grew. “yl2.1” seedlings showed reduced growth and lethality during the fourth leaf stage [21]. In tomatoes, Dechkrong and colleagues obtained a variegated leaf color mutant through EMS treatment, with the cotyledons consistent with the wild type, but the true leaves exhibited deep green (DG), medium green (MG), light green (LG), and white (WH) patterns [22]. In peppers, Arisha and his research group identified a yellow leaf mutant [15]. Radiation, as the fundamental method of physical mutagenesis, has also been used in the study of vegetable leaf color mutants. For example, Yang and collaborators obtained a mutant “R24” through radiation mutagenesis in peppers, which displayed yellow leaves throughout its entire growth period [14]. Li and his team acquired a cabbage yellow leaf mutant using *60*Coγ-ray [23]. In recent years, T-DNA insertion and transposon introduction have gradually been applied to the construction of mutant libraries. Huang and colleagues screened an Arabidopsis albino mutant through T-DNA insertion [24].

## 3. Localization of Leaf Color Mutant Genes in Vegetables

### 3.1. Map-Based Cloning

Map-based cloning, also known as positional cloning, is a cloning technology that leverages the chromosomal location of the target gene. This method involves creating extreme pools of the target trait within segregating populations and employs molecular marker technologies such as RFLP markers based on Southern blot hybridization, RAPD and SSR markers based on PCR amplification, and SNP and Indel markers based on high-throughput sequencing and DNA chip technology. RFLP (Restriction Fragment Length Polymorphism): RFLP is one of the earliest molecular marker techniques, known for its high resolution. However, it is labor-intensive and less modern compared to newer technologies. It involves digesting DNA with restriction enzymes and analyzing fragment lengths through gel electrophoresis. SSR (Simple Sequence Repeat): SSRs, also known as microsatellites, are highly polymorphic markers widely used in genetic studies. They are particularly suitable for breeding programs and genetic mapping due to their high variability and ease of use. SNP (Single Nucleotide Polymorphism)**: SNPs represent a modern approach to genetic markers, characterized by high density and high throughput. They are ideal for genome-wide association studies (GWASs) and fine mapping due to their ability to capture genetic variation at a detailed level. AFLP (Amplified Fragment Length Polymorphism): AFLP is a high-throughput technique suitable for generating a large number of markers. It combines elements of RFLP and PCR to produce a large set of DNA fragments for analysis, making it useful for large-scale marker generation. InDel (Insertion/Deletion): InDels are simple markers that involve insertions or deletions in the DNA sequence. They are useful for targeted analysis and are particularly effective for studying specific genes due to their straightforward detection methods. APS (Cleaved Amplified Polymorphic Sequence): CAPS markers are simple and targeted, similar to InDels. They involve detecting restriction enzyme sites within PCR-amplified DNA fragments and are useful for analyzing specific genes. ach marker technology has its own strengths and weaknesses, and the choice of marker depends on the specific requirements of the study or breeding program. It begins with selecting polymorphic molecular markers from both parents for initial gene positioning. Following the initial gene interval positioning, the population size is expanded, and fine mapping is conducted through flanking molecular markers and high-density molecular linkage analysis. Currently, positional cloning technology has been extensively applied in the localization of leaf color mutation genes. Zha and colleagues identified the cucumber yellow leaf mutant “yl”, which exhibited a significant reduction in chlorophyll content, an abnormal chloroplast ultrastructure, and decreased photosynthetic capacity compared to the wild type. By crossing the “yl” mutant with Hazerd, they obtained 245 F_2_ mapping populations and located the “yl” locus between the yb-Indel3-2 and yb-Indel3-3 markers on chromosome 3, approximately 900 kb apart. Using nine polymorphic InDel markers within this region, the genotyping of 737 F_2_ individuals led to the identification of the candidate gene *CsaV3_3G009150*, encoding the chloroplast signal recognition particle receptor cpFtsY, within a 100 kb genomic region [25]. Additionally, Zhang and others determined the candidate gene *CsSRP43*, encoding the chloroplast signal recognition particle 43 protein, through positional cloning in the cucumber yellow leaf mutant “yf” [26]. In cabbage, Zhao and colleagues identified the mutant “yvl”, which displayed light yellow leaves during the cotyledon stage, gradually turning green at maturity. Using developed molecular markers, the “yvl” locus was mapped to a 70 kb region between the markers yvl-O10 and InDel-O6. Screening for co-segregating SNP markers with the “yvl” phenotype identified *BnaA03g04440D* as a potential candidate gene believed to be involved in chlorophyll biosynthesis and plastid-to-nucleus signaling [19]. In the cabbage virescent mutant *cde1*, Yang and colleagues used SSR markers to finely map the *BnCDE1* locus to an area of 175.55 kb between BnC08A197 and BnC08A741 [27]. In soybeans, Liu and colleagues identified a yellow leaf phenotype mutant controlled by two homologous genes, *YL1* and *YL2*. Between the SSR markers BRACSOYSSR_11_0156 and BRACSOYSSR_11_0175 on chromosome 11, they found the candidate gene *Gly-ma11g04660* for the *YL1* locus. Based on this locus, they cloned the homologous *YL2* candidate gene, identifying the candidate gene *Glyma01g40650*. *YL1* and *YL2* are involved in the regulation of light absorption during photosynthesis [13].

### 3.2. BSA-Seq

BSA-Seq is a gene mapping technique that integrates Bulked Segregant Analysis (BSA) with Next Generation Sequencing (NGS) technologies. Initially proposed by Michelmore, it was utilized in the localization of resistance genes for downy mildew in lettuce [28]. With the rapid progress of high-throughput sequencing technology, BSA-Seq has become increasingly popular for the localization of genes associated with vegetable leaf color mutations. Compared to other methods, BSA-Seq offers precise and efficient identification of target genes and enables rapid screening of candidate genes based on resequencing data. A new virescent leaf mutant 104Y, identified by spontaneous mutation, was studied. The mutant’s cotyledons and upper five true leaves are yellow, gradually turning green with increased chlorophyll content. Genetic analysis mapped the trait to a single recessive gene, v-2, located on chromosome 3 (Table 1). Fine mapping narrowed v-2 to a 73 kb region containing eight genes. BSA-seq and cDNA sequencing identified a nonsynonymous mutation in the Csa3G890020 gene, encoding an auxin F-box protein, as the likely candidate gene. Transcriptome and qPCR analyses showed no change in Csa3G890020 expression but revealed down-regulation of key genes involved in chlorophyll biosynthesis and auxin signaling in 104Y compared to EC1, suggesting post-transcriptional regulation. This study is the first to clone an auxin F-box protein gene related to virescent leaves in cucumber, providing new insights into chlorophyll biosynthesis regulated by auxin signaling [20].

#### 3.2.1. QTL-Seq

With the swift progress in second-generation sequencing technology, Takagi introduced the QTL-Seq method. This approach capitalizes on extreme phenotypes from a segregating population to form bulks, performs whole-genome resequencing of these bulks, analyzes the SNPs within them, and calculates the SNP-index value for each SNP. When the ∆(SNP-index) value for a chromosome segment surpasses a predetermined threshold, this indicates the presence of a QTL at that location. Compared to conventional QTL mapping techniques, QTL-Seq is more efficient and precise. Currently, QTL-Seq has proven effective in the genetic mapping of various vegetable leaf color mutants. In cucumbers, Pan selected 25 mutants and wild-type plants to construct bulks for sequencing. Through comparison with the reference genome, the mutant gene *Csyc* locus was mapped to physical positions between 22.90 and 23.24 Mb and between 26.56 and 27.23 Mb on Chr3 (Table 1). After linkage analysis and resequencing, *Csyc* was localized to a 96.8 kb region between SNP23167277 and SNP23264091. Moreover, SNP23218243 (T to C) was identified in the promoter region of the potential candidate gene *CsaV3_3G026880*, which encodes a YSL transporter [29]. Zhou identified a light green true leaf mutant *se59* and performed resequencing of the double bulks. They screened *CsaV3_3G016210* containing SNP 12112564 as the candidate gene for *se59*. This SNP locus is located in the first exon of *CsaV3_3G016210*, and the mutation results in an amino acid change in the gene. *CsaV3_3G016210* encodes an invertase/pectin methylesterase inhibitor (INV/PMEI) [30]. Zhang discovered a spontaneous cucumber mutant *SC311Y* with yellow cotyledons and true leaves that gradually turn green. Using the boundary molecular markers UW084839 and SSR15124, they identified the candidate gene *Csa3G042730* within the 33.54~35.66 Mb interval on chromosome 3 (Table 1). This candidate gene may regulate chloroplast development through the regulation of Plastid Division 2 (PDV2) [31]. Lin localized the candidate gene for the cabbage yellow leaf mutant in proximity to the SSR marker bna108, between 3.36 Mb and 6.07 Mb on chromosome A01 (Table 1), via BSA-Seq [32].

#### 3.2.2. Mut-Map

Mut-Map is a genetic breeding analysis technology that relies on the existing reference genome of the parents. As sequencing technology has advanced and costs have decreased, an increasing number of crops have undergone whole-genome sequencing. High-quality reference genomes have laid a solid foundation for the cloning of key trait genes, the investigation of molecular mechanisms, and the genetic improvement of crops [33], which has facilitated the widespread application of Mut-Map. Mut-Map is primarily used for gene mapping in mutants produced by EMS mutagenesis. EMS mutagenesis typically induces single-base mutations. By backcrossing the mutant with the wild type used for mutagenesis, followed by self-crossing, mutant phenotype bulks and wild-type phenotype bulks are constructed within the F_2_ generation population. Subsequently, resequencing analysis is conducted, and SNP molecular markers are used for gene mapping of the target trait. Abe initially determined the genomic locations of seven mutant genes responsible for significant agronomic trait mutations in a rice variety through Mut-Map [34]. Since then, Mut-Map has been widely reported in leaf color mutants of vegetables. In cucumbers, Cao identified a variegated leaf mutant *Csvl* that showed a considerable reduction in plant height and leaf area, abnormal chloroplast structural characteristics, and a marked decrease in photosynthetic pigment content and photosynthetic rate compared to the wild type. The mutant and wild type were crossed, and Mut-Map was used to resequence the DNA of a bulk of 17 F_2_ plants with the variegated leaf phenotype and one wild-type plant. A base mutation was discovered at SNP 18277305, which is located on a chorismate synthase gene (Table 1) [35]. In Chinese cabbage, Wang discovered an evergreen mutant *nym1* that maintained its green color throughout the leaf senescence process. Mut-Map localized the candidate gene to a 1.59 Mb region between 24,908,848 and 26,503,461 on chromosome A03 (Table 1). Genotype analysis using SNPs predicted that *BraA03g050600.3C*, which encodes a magnesium chelatase, is the candidate gene for *Brnym1* [36]. Based on Bulked Segregant Analysis using Mut-Map, Dechkrong found that the FtsH-like protein precursor located on chromosome 4 is most likely the candidate gene for the tomato variegated leaf mutant [22].

### 3.3. Transcriptome Sequencing

The transcriptome encompasses a collection of coding and non-coding RNAs. In gene mapping, transcriptome sequencing typically leverages high-throughput sequencing technology to screen for genes associated with target traits by analyzing differentially expressed genes between various samples. Currently, RNA-Seq has been extensively employed in research to uncover leaf color mutation genes in vegetables. Guo isolated a dark green leaf mutant *GL* from the tomato inbred line “716”, which exhibited a significantly higher chlorophyll content and photosynthetic rate than the wild type. Additionally, the mutant’s mesophyll tissue was more developed, with a tighter arrangement of palisade cells. Transcriptome analysis of the mutant and wild type identified a total of 131 upregulated differentially expressed genes (DEGs) and 161 downregulated DEGs. Notably, the expression levels of the photosynthetic antenna gene *Solyc02g071030* (*LHCB1*) and diterpenoid biosynthesis-related genes *Solyc08g005710* and *Solyc09g059240* were significantly higher in *GL* leaves compared to WT leaves. This suggests that the leaf color mutation is related to the biosynthesis of diterpenoids and photosynthetic pathways [17]. Huo conducted research on the yellow cotyledon mutant *19YC-2* of Chinese cabbage and found that the cotyledons of *19YC-2* displayed a distinct yellow color during the cotyledon stage, turning green from the two-leaf stage onward. The first leaf appeared yellow during the two-leaf stage but gradually turned green by the four-leaf stage. Resequencing analysis of population samples from the cotyledons and the fourth leaves of both the mutant and wild type revealed that DEGs encoding photosynthetic antenna proteins and carotenoid biosynthesis genes with differential expression may play crucial roles in regulating the leaf color changes in the mutant [37]. Yan reported a photosensitive albinism mutant *alc* in cucumber, which showed white cotyledons under normal light conditions and developed cream-green cotyledons under low light conditions, but failed to produce true leaves. Compared to the well-developed chloroplasts in the wild-type cotyledons, the number of chloroplasts in the mutant was significantly reduced. Transcriptome analysis revealed that genes involved in chlorophyll metabolism and the methylerythritol 4-phosphate (MEP) pathway were affected in the *alc* mutant [16].

## 4. Molecular Mechanism of Leaf Color Mutation in Vegetables

### 4.1. Chloroplast Development Pathway

In horticultural crops, mesophyll cells are the main sites where photosynthesis occurs, and chloroplasts are the key organelles responsible for this process. The content of chloroplasts in plants is primarily regulated by their biosynthesis. However, if the chloroplast content in mesophyll cells is irregular, this can lead to various leaf color mutations in plants. Chloroplast development is controlled by a complex genetic network that includes numerous light signals and hormone signals.

#### 4.1.1. Photomorphogenesis

**Figure 1:**
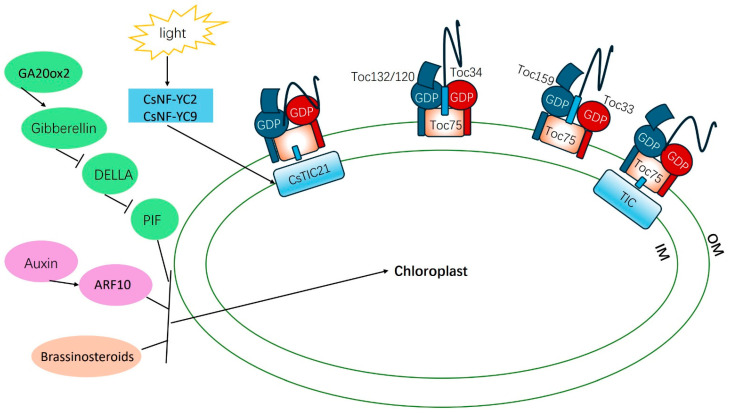
The main pathways of chloroplast biosynthesis.

Light is an essential factor for chloroplast development in angiosperms. Light signal receptors are capable of receiving light signals and undergoing conformational changes, which then regulate the expression of downstream genes through interactions, thus controlling chloroplast development. The majority of light signal receptors act as transcription factors, and phytochromes and cryptochromes, which detect red/far-red and blue light, respectively, are considered particularly significant among these receptors. In the cytoplasm, phytochromes are converted into their Pfr-active form upon sensing red light and then translocate to the nucleus. Once in the nucleus, they interact with various transcription factors, including phytochrome-interacting factors (PIFs), to regulate the expression of genes related to chloroplast development. In a study reported by Ke, the functional loss of the candidate gene *CsTIC21* in the cucumber albinism mutant resulted in the formation of malformed chloroplasts. This gene plays a role in photomorphogenesis, and the light signal transcription factors *CsNF-YC2* and *CsNF-YC9* specifically bind to the *CsTIC21* promoter and enhance gene transcription, thereby participating in chloroplast development [38] (Figure 1).

#### 4.1.2. Hormone Signaling

Hormone signals are also believed to play a role in regulating chloroplast development. For instance, endogenous hormones such as gibberellins (GA), brassinosteroids (BRs), and indole-3-acetic acid (IAA) participate in the signaling network of chloroplast development. They are triggered or activated by light signals to initiate downstream signaling. The darkening of leaf color is a significant phenotype resulting from a deficiency of gibberellins in horticultural crops. It has been discovered that PIF, a key regulatory factor in chloroplast development, is inhibited by DELLA, a negative regulatory factor of the GA signal. Mutants related to GA synthesis, such as *GA20ox2*, also exhibit partial de-greening [39]. The first brassinosteroid-related mutant identified to be linked with chloroplast development was *det2*, which shows dark green leaves, delayed senescence, and early flowering when grown under light conditions [40]. Additionally, in tomatoes, the auxin response factor *ARF10* has been found to be involved in the regulation of chloroplast development (Figure 1).

#### 4.1.3. Nuclear Protein Transport and Chloroplast Protein

Nearly 3000 proteins in chloroplasts are encoded by the nucleus, synthesized as precursor proteins in the cytoplasm and subsequently transported into the chloroplast. These genes play crucial roles in chloroplast biosynthesis, metabolite transport, and other processes. Among them, protein transmembrane transport facilitated by the *TOC-TIC* complex is vital for maintaining the stability of chloroplast proteins. The Toc159 protein subunit in Arabidopsis is encoded by four genes: *AtTOC159*, *AtTOC132*, *AtTOC120*, and *AtTOC90*. In a study conducted by Bauer, the Arabidopsis albinism mutant toc159 displayed an albino phenotype [41].

Although chloroplasts contain a multitude of nuclear-encoded genes, chloroplast development is actually governed by a combination of nuclear and chloroplast-encoded genes. Plant chloroplasts encode about 80 proteins, among which the PEP (plastid-encoded RNA polymerase) complex has been shown to play an essential role in chloroplast development. Research by Gao demonstrated that in tomato leaf color mutants, the transcription of chloroplast-encoded PEP-dependent genes such as *PsaB*, *PsaA*, *PsbA*, and *PsbB* was inhibited. This inhibition led to significantly reduced expression of *WV* genes, blocking chloroplast differentiation and chlorophyll synthesis and resulting in a white-green leaf phenotype [42]. Similarly, in Brassica napus, CHLH limits PEP activity, leading to the degradation of chloroplast structure and chlorophyll biosynthesis. This results in a pale yellow appearance of young leaves in mutant plants during the cotyledon stage, which gradually turn light green as they mature [19].

#### 4.1.4. Chloroplast Division

Chloroplasts increase their numbers in plant cells through secondary division, a process that largely depends on their division machinery. This initiation apparatus is located at the center of the chloroplast and includes an FtsZ ring on the stromal side of the inner membrane and an ARC5 ring on the cytoplasmic side of the outer membrane. The FtsZ ring is primarily composed of FtsZ1 and FtsZ2 proteins, while the ARC5 ring is predominantly formed by the recruitment of plastid division proteins 1/2 (PDV1/PDV2). Current research has revealed that in Arabidopsis and Physcomitrella patens, the overexpression of PDV proteins leads to an increase in the number of chloroplasts and a reduction in their size. Furthermore, during leaf development, the rate of chloroplast division decreases as PDV protein levels diminish, while the levels of other division components remain unchanged. Consequently, PDV acts as a regulator in the process of chloroplast division [43].

### 4.2. Chlorophyll Biosynthesis Pathway

The synthesis of chlorophyll is typically divided into three stages: the synthesis of 5-aminolevulinic acid, the processing of 5-aminolevulinic acid to Mg-protoporphyrin, and the synthesis of Mg-protoporphyrin into chlorophyll a and b. The chlorophyll precursor, aminolevulinic acid, is synthesized through these three stages, transforming into chlorophyll through the action of 18 key enzymes encoded by over 30 genes. This sequence of reactions primarily occurs in three distinct locations: the chloroplast stroma, the chloroplast membrane, and the thylakoid membrane. The first reaction in chlorophyll synthesis takes place in the chloroplast stroma, where L-glutamyl-tRNA is catalyzed by glutamyl-tRNA synthetase to form 5-aminolevulinic acid. Following this, a series of reactions from 5-aminolevulinic acid to protoporphyrin IX synthesis occurs in the chloroplast stroma, with protoporphyrin being converted into Mg-protoporphyrin on the chloroplast membrane through the action of magnesium chelatase. Under the influence of divinyl reductase, divinyl chlorophyllide a is transformed into chlorophyllide a and divinyl chlorophyllide b. Finally, chlorophyll a and chlorophyll b are synthesized on the thylakoid membrane. Numerous leaf color mutants have been identified due to mutations in genes associated with this biosynthetic pathway, resulting in inhibited chlorophyll synthesis. Moreover, mutations in genes located further upstream in the pathway are more likely to produce bleaching or yellowing phenotypes. Glutamyl-tRNA reductase and magnesium chelatase are crucial regulatory points in this biosynthetic pathway, playing a key role in chlorophyll synthesis. Glutamyl-tRNA reductase is encoded by the HEMA gene family. Liu found that in rice leaf color mutants, the number, morphology, and physiology of chloroplasts and mitochondria were severely affected. They further isolated and cloned the gene *OsGluRs*, which encodes glutamyl-tRNA reductase, and this gene was responsible for the production of a yellow-green leaf color. Therefore, glutamyl-tRNA synthetase may play an important role in the proliferation, development, and physiology of chloroplasts and mitochondria [44]. Magnesium chelatase is composed of three subunits: ChlI, ChlD, and ChlH. Gao identified a chlorophyll-deficient mutant, *C528*, in cucumber that displayed a golden-yellow leaf color throughout development. They determined that the *CsChlI* gene, encoding the CHLI subunit of cucumber magnesium chelatase, contained the mutation. Furthermore, the mutation occurred in the highly conserved nucleotide binding region of the CHLI protein, an area where chlorophyll-deficiency mutations are frequently found [45].

### 4.3. Genes Related to the Heme–Phytochrome Metabolic Pathway

In the photosignal transduction pathway of plants, phytochrome is generated from heme through the action of heme oxygenase, which converts heme into biliverdin and eventually synthesizes the phytochrome chromophore. The synthesis of protoporphyrin shares a pathway with chlorophyll from the synthesis of 5-aminolevulinic acid (ALA) to the processing of ALA to protoporphyrin IX. The key distinction lies in the fact that protoporphyrin IX, when complexed with a magnesium ion, forms magnesium protoporphyrin and is synthesized into chlorophyll, whereas when complexed with an iron ion, it transforms into heme. The accumulation of heme exerts negative feedback regulation on the content of 5-aminolevulinic acid. Phytochrome oxygenase controls the content of heme, thereby influencing 5-aminolevulinic acid and subsequently affecting chlorophyll synthesis. In tomatoes, Terry discovered leaf yellowing mutants *au* and *yg-2*, which resulted from mutations in the genes encoding heme oxygenase and phytochrome chromophore synthase. These mutations cause an accumulation of heme, inhibiting the synthesis of the common precursor of chlorophyll and heme, ALA, in the plant. This impedes the normal pathway of chlorophyll synthesis, leading to decreased chlorophyll content [46].

### 4.4. Chlorophyll Degradation Pathway

In plant cells, the degradation and synthesis of chlorophyll occur simultaneously, with the degradation process primarily taking place in aging chloroplasts and vacuoles. This equilibrium alongside the chlorophyll synthesis process controls the chlorophyll content within the plant. Within the chloroplasts, chlorophyll is broken down into primary fluorescent chlorophyll metabolites under the action of chlorophyllase, dechelatase, dechelating chlorophyllase, and chlorophyll metabolic product reductase. In vacuoles, the acidic conditions catalyze the transformation of primary fluorescent chlorophyll catabolites into non-fluorescent chlorophyll catabolites by enzymes. Mutations in the chlorophyll degradation pathway often result in the phenotype for the leaf retention of chlorophyll. Studies have shown that in rice, the *Sgr* gene encodes a chloroplast protein that interacts with LHCPII in the chloroplast, thereby regulating leaf aging. Consequently, mutations in this gene in the mutant *sgr* impede the chlorophyll degradation pathway, leading to the leaf retention phenotype observed in *sgr* [47]. Furthermore, Wang identified a variegated mutant *nye* in cabbage, where mutations in the controlling gene *Brnye1* prevent the conversion process from chlorophyll b to chlorophyll a, resulting in the observed phenotype [48].

## 5. Interaction Mechanisms Between Environmental and Genetic Factors in Regulating Leaf Color Development in Vegetables

Environmental factors significantly impact leaf color variations in vegetables. Understanding the interaction mechanisms between environmental and genetic factors is crucial for developing high-quality vegetable varieties. Light affects chlorophyll synthesis and chloroplast development, impacting leaf color. High light intensity enhances chlorophyll content, leading to darker green leaves, while low light intensity results in lighter green or yellow leaves. Specific wavelengths (e.g., red and blue light) activate photoreceptors that regulate genes related to chloroplast development and chlorophyll synthesis. Temperature influences the activity of enzymes involved in chlorophyll biosynthesis. Higher temperatures accelerate chlorophyll degradation, causing yellowing of leaves, while lower temperatures slow down chlorophyll synthesis, resulting in lighter green leaves. Temperature also affects the expression of genes related to chloroplast development, such as those encoding heat shock proteins and cold-responsive proteins. Nutrients like nitrogen and magnesium are essential for chlorophyll synthesis. Nitrogen deficiency reduces chlorophyll content and causes yellowing of leaves, while magnesium deficiency impairs chlorophyll stability. Nutrient imbalances can also affect the expression of genes involved in nutrient uptake and chlorophyll biosynthesis pathways. Hormones such as gibberellins (GAs) and brassinosteroids (BRs) interact with environmental factors to regulate leaf color. GAs promote chlorophyll synthesis and chloroplast development, while BRs enhance photosynthetic efficiency. Environmental stresses like drought and high salinity can alter hormone levels, affecting leaf color. Environmental stresses (e.g., drought, high salinity, extreme temperatures) induce reactive oxygen species (ROS) production, damaging chloroplasts and causing chlorophyll degradation. Plants respond to these stresses by activating antioxidant enzymes and expressing stress-responsive genes to protect chloroplasts and maintain leaf color. The interaction between genetic and environmental factors is complex. Gene expression related to chlorophyll biosynthesis and chloroplast development is regulated by both genetic and environmental signals. Mutations in these genes can alter the plant’s response to environmental factors, leading to variations in leaf color under different conditions.

## 6. Breeding Applications of Leaf Color Mutants

Leaf color mutants offer significant potential for enhancing yield, quality, and stress resistance in vegetable breeding. Here, we outline key applications.

### 6.1. Enhancing Photosynthetic Efficiency

Leaf color mutants with higher chlorophyll content or improved chloroplast development can boost photosynthetic efficiency. For instance, the tomato mutant GL, with elevated chlorophyll levels, shows enhanced photosynthesis and higher yield potential compared to wild types [17]. Similarly, identifying genes like CsTIC21 in cucumber can lead to varieties with better photosynthetic performance [38].

### 6.2. Developing Stress-Resistant Varieties

Mutants with enhanced chlorophyll content often exhibit better tolerance to environmental stresses such as drought and high light. For example, an albino mutant in Arabidopsis identified by Huang et al. [24] can serve as a model for understanding stress resistance. Transferring relevant genes into vegetable crops can help develop stress-resistant varieties.

### 6.3. Improving Nutritional Quality

Leaf color mutants can enhance the nutritional quality of vegetables. Mutants with higher chlorophyll content may also have elevated levels of beneficial compounds like carotenoids and flavonoids. Identifying genes involved in chlorophyll biosynthesis, such as CsChlI in cucumber [45], can lead to varieties with improved nutritional profiles.

### 6.4. Ornamental and Specialty Varieties

Leaf color mutants can be used to develop visually appealing ornamental and specialty vegetable varieties. Mutants with unique colors like yellow, red, or variegated patterns can meet the growing market demand for specialty vegetables. For example, the yellow cotyledon mutant in Chinese cabbage studied by Huo et al. [37] provides insights into leaf color variation, which can be applied to develop ornamental varieties.

### 6.5. Marker-Assisted Selection

Leaf color mutants can serve as valuable markers for marker-assisted selection (MAS). Identifying specific molecular markers linked to leaf color genes, such as the InDel markers used in the fine mapping of the cucumber mutant “yl” [25], can accelerate the breeding process and improve the efficiency of developing new varieties.

### 6.6. Case Studies

Several successful breeding cases demonstrate the potential of leaf color mutants. For example, using EMS mutagenesis to create yellow leaf mutants in cucumber [18] and cabbage [19] has led to the identification of key genes involved in chlorophyll biosynthesis. These genes can be targeted to develop new varieties with desired leaf color traits. Another example is the identification of the FtsH-like protein precursor in tomato, which can be used to develop varieties with unique leaf patterns [22].

In conclusion, leaf color mutants provide significant potential for vegetable breeding. By leveraging genetic and molecular insights, new vegetable varieties with enhanced yield, quality, and stress resistance can be developed. Further exploration of specific breeding cases and application strategies is needed to fully utilize the genetic resources of leaf color variation in breeding programs.

## 7. Prospects

Leaf color mutants are widely distributed and easily identifiable. Leaf color traits not only affect the stress resistance and nutritional quality of vegetables but also have certain impacts on developmental regulation and other aspects. Moreover, vegetable leaf color mutants hold significant potential in high-quality breeding programs. Additionally, as the value of ornamental vegetables continues to rise, cultivating edible vegetable varieties with diverse leaf colors using leaf color mutants can significantly boost the economic value of vegetables. Currently, research on vegetable leaf color mutants primarily focuses on agronomic traits, photosynthetic characteristics, and molecular mechanisms. The molecular mechanisms behind vegetable leaf color mutations involve complex regulations across multiple biological pathways. Therefore, as more genes related to vegetable leaf color mutations are identified and characterized, there is a need to continuously deepen research on the structure and function of the photosynthetic system in vegetable crops, as well as its regulatory mechanisms. At present, leaf color mutants are widely used in studies on mechanisms and underlying processes but less frequently in breeding applications. With the continuous progress in molecular biology and functional genomics, clarifying the color-changing mechanisms of leaf color mutants will facilitate the application of leaf color mutant materials in breeding practices. In-depth studies into the relevant molecular mechanisms also provide abundant genetic resources and utilization value for leaf color mutant traits in the high-quality breeding of vegetable crops.

**Table 1:** Studies on the localization of reported leaf color mutants in vegetables.

Reference	Species	Source	Localization	The Localization on Chromosomes	Gene Name	Gene Number
[5]	*Cucumis sativus* L.	EMS	BSA-Seq Fine mapping	Within approximately 45.3 kb of the region defined by the two markers CAPS777-1 and Indel777-3 on chromosome 3	*CsHD*	*Csa3G836480*
[18]	*Cucumis sativus* L.	EMS	BSA-Seq Fine mapping	Within the 86.3 kb region between the molecular markers UW804200 and SSR05515 on chromosome 4	*CsVYL*	*Csa4G637110*
[20]	*Cucumis sativus* L.	EMS	BSA-Seq Fine mapping	Within the 73kb region between the molecular markers NSN and SNP16 on chromosome 3	*v-2*	*Csa3G890020*
[30]	*Cucumis sativus* L.	EMS	BSA-Seq	Locus SNP12112564 on chromosome 3	*CsSE59*	*CsaV3_3G016210*
[6]	*Cucumis sativus* L.	-	Fine mapping	The 50.4 kb region between the molecular markers v1SSR8 and CAPs15 on chromosome 6	*v-1*	*CsaCNGCs*
[7]	*Cucumis sativus* L.	Spontaneous	Whole-genome resequencing	The interval defined by the molecular markers SNP11124523-SNP11216771 on chromosome 4	*ygl1*	*Csa4M286960* *Csa4M287550* *Csa4M288070* *Csa4M288080*
[21]	*Cucumis sativus* L.	EMS	BSA-Seq Fine mapping	Within the 167kb region defined by the molecular markers Indel22 and SNP81 on chromosome 2	*CsYL2.1*	*Csa2G263900*
[25]	*Cucumis sativus* L.	EMS	BSA-Seq Fine mapping	Within the 100 Kb region defined by the molecular markers AInd3-16 and AInd3-24 on chromosome 3	*Cscpftsy*	*CsaV3_3G009150*
[26]	*Cucumis sativus* L.	Spontaneous	Fine mapping whole-genome sequencing	The interval defined by the molecular markers InDel8 and SSR20583 on chromosome 7	*CsSRP43*	*CsGy7G001220*
[31]	*Cucumis sativus* L.	Spontaneous	BSA-Seq Fine mapping RNA-Seq	Within the region defined by the molecular markers UW084839 and SSR15124 on chromosome 3	*v-3*	*Csa3G042730*
[38]	*Cucumis sativus* L.	EMS	Map-based cloning BSA-Seq	Within the 63.44Kb interval defined by the molecular markers SBP7349 and SNP0787 of chromosome 7	*CsTIC21*	*Csa7G071680*
[35]	*Cucumis sativus* L.	EMS	BSA-Seq RNA-Seq	Chromosome 6 molecular marker SNP-18277305	*Cscs*	*Csa6G405290*
[36]	*Brassica campestris* L.	EMS	MutMap	Chromosome A03	*Brnym1*	*BraA03g050600.3C*
[12]	*Brassica rapa* L.	EMS	BSR-Seq Fine mapping whole-genome resequencing	Within the 64.25 kb region defined by the molecular markers INDEL-N14 and INDEL-I8 of chromosome A10	*Brpem1* *Brpem2*	*BraA10g021490.3C* *BraA10g021490.3C*
[19]	*Brassica napus*	EMS	BSA-Seq Map-based cloning RNA-Seq	Within the 70kb interval defined by the molecular markers yvl-O10 and InDel-O6 of chromosome A03	*BnaA03.CHLH*	*BnaA03g04440D*
[28]	*Brassica napus*	EMS	Fine mapping		*BnCDE1*	*BnaC08g34840D*
[22]	*Solanum lycopersicum*	EMS	whole-genome sequencing MutMap	Locus NC_015441.3 of chromosome 4	*FtsH-like protein precursor*	*LOC100037730*
[13]	*Glycine max*	Spontaneous	BSA-Seq Map-based cloning	YL1 is located at chromosome 11 (within the 270kb range defined by the markers BARCSOYSSR_11_0156 and BARCSOYSSR_11_0175); YL2 is located in chromosome 1 (within the 270kb interval defined by BARCSOYSSR_11_0164 and BARCSOYSSR_11_0169).	*YL1* *YL2*	*glyma11g04660* *glyma01g40650*
[14]	*Capsicum annuum* L.	^60^Co γ-ray	Fine mapping	The 214 kb region defined by the molecular markers SNP5791587 to SNP6011215 on chromosome 9	*CaLY1*	*Capana09g000166*
[48]	*Brassica campestris* L.	Spontaneous	BSA-Seq Fine mapping	Within the 81.01kb region defined by the molecular markers SSRWN27 and SSRWN30 of chromosome A03	*Brnye1*	*Bra019346*

## Data Availability

Data are contained within the article.
